# Ambidexterity

**Published:** 1904-03-26

**Authors:** 


### AMBIDEXTERITY.

We have much pleasure in calling attention to a very useful society for the promotion of ambidexterity, which is holding meetings and discussing papers at the house of the Medical Society of London, and which is calculated, we think, to be of much benefit to the community. A great many years have passed away since Benjamin Franklin first struck the note of common sense upon this subject in the humorous essay which he entitled, "The complaint of a Neglected Half-brother." In this complaint the left hand was made to say that he had been born with capabilities fully equal to those of the right, but that he had not only never been taught a trade or assisted towards earning his own living, but that he had even been almost uniformly chidden and discouraged in his attempts. The truth of this is common knowledge. A child at table who took his knife in his left hand and his fork in his right, or a child at school who took up his pen or pencil with his left hand, would be reproved by the great majority of both parents and teachers. The result is that the left hands of the majority of adults have grown up in a state of very limited usefulness and of comparative weakness and awkwardness, while the motor centres supplying them have been left in a partially-developed condition. That this state of things is purely artificial admits of easy proof from the experience of all those who have educated the left hand systematically; and there can be no doubt that the consequently increased blood supply which is directed to the right motor centres of the brain has a beneficial effect upon the nutrition of that organ generally, and tends to the promotion of intelligence as well as to the establishment of ambidexterity. The power of using the left hand has often been found valuable by surgeons, and the attainment of this power has, from remote times, been recommended in many treatises. It is, of course, of especial value in ophthalmic surgery, since operators who are only right handed are compelled to place themselves in a variety of awkward positions in order to use instruments upon the left eye, the accessibility of which is diminished from the right side by the prominence of the nose. Ambidextrous swordsmen have long been known to be specially dangerous adversaries; and are the special products of the best Swedish schools of fencing. In quite recent times a few American teachers have been pioneers in the way of education in general ambidexterity, and the excellent results which they have obtained are now being sought for by a few in our own country, to whose endeavours we wish every possible success. The first step towards the necessary training is accomplished at the drawing board; and, in a few years, complete equality and interchangeability of the hands may be obtained by the great majority of pupils; while, in a few, the left hand becomes definitely the better of the two. It is curious to see from the many autographs of Lord Nelson which have been preserved, how much better he wrote with his left hand than he had written originally with his right; and the same experience would not, we believe, be at all uncommon. The few objections that have been raised from time to time against the proposed routine education of the left hand are for the most part of quite a trivial nature, and the only attempt at opposition based on scientific principles, so far as we know, is to be derived from Professor Lombroso's statistics, according to which the percentage of individuals who are left handed is from three to five times as high in criminals as in the more respectable members of society. But the numbers upon which Professor Lombroso establishes his statistics are too small to found a scientific conclusion upon.

We hope for the complete success of the young Society, and feel sure that the general adoption of ambidextrous training would be of unmixed benefit to the community.

